# Investigation on energy bandgap states of amorphous SiZnSnO thin films

**DOI:** 10.1038/s41598-019-55807-2

**Published:** 2019-12-17

**Authors:** Byeong Hyeon Lee, Kyung-Sang Cho, Doo-Yong Lee, Ahrum Sohn, Ji Ye Lee, Hyuck Choo, Sungkyun Park, Sang-Woo Kim, Sangsig Kim, Sang Yeol Lee

**Affiliations:** 1 Department of Microdevice Engineering, Korea University, Seoul, 136-701 South Korea; 2 Imaging Device Laboratory, Samsung Advanced Institute of Technology, Suwon, 16678 South Korea; 3 Department of Physics, Pusan National University, Busan, 609-735 South Korea; 4 School of Advanced Materials Science and Engineering, Sungkyunkwan University, Suwon, 16419 South Korea; 5 Department of Semiconductor Engineering, Cheongju University, Cheongju, 28503 South Korea; 6 Research Institute of Advanced Semiconductor Convergence Technology, Cheongju, 28503 South Korea

**Keywords:** Engineering, Materials science

## Abstract

The variation in energy bandgaps of amorphous oxide semiconducting SiZnSnO (a-SZTO) has been investigated by controlling the oxygen partial pressure (*O*_*p*_). The systematic change in *O*_*p*_ during deposition has been used to control the electrical characteristics and energy bandgap of a-SZTO. As *O*_*p*_ increased, the electrical properties degraded, while the energy bandgap increased systematically. This is mainly due to the change in the oxygen vacancy inside the a-SZTO thin film by controlling *O*_*p*_. Changes in oxygen vacancies have been observed by using X-ray photoelectron spectroscopy (XPS) and investigated by analyzing the variation in density of states (DOS) inside the energy bandgaps. In addition, energy bandgap parameters, such as valence band level, Fermi level, and energy bandgap, were extracted by using ultraviolet photoelectron spectroscopy, Kelvin probe force microscopy, and high-resolution electron energy loss spectroscopy. As a result, it was confirmed that the difference between the conduction band minimum and the Fermi level in the energy bandgap increased systematically as *O*_*p*_ increases. This shows good agreement with the measured results of XPS and DOS analyses.

## Introduction

In recent decades, transparent amorphous oxide semiconductors (TAOSs) have been studied in various fields, such as optoelectronics, active-matrix displays, and integrated circuits^1–3^. The most important advantage of TAOS is that it can provide high field-effect mobility even though it is amorphous, and it can be applied to transparent electronic devices because it has high transmittance in the visible light region^4^. This is because the energy bandgap of the oxide semiconductor is known to be greater than 3 eV, and the outermost orbital of the metal cation is composed of spherical S-orbitals; therefore, the movement of electrons overlap smoothly^2^. In addition, TAOS can be applied to a wide range of industrial fabrication methods. For example, sol-gel^5^, pulse layer deposition^6^, magnetron sputtering^7^, chemical vapor deposition^8^, and atomic layer deposition^7^ are known to be very attractive materials with no significant limitations on their deposition methods. These TAOS can be designed in various configurations from binary material (ex. ZnO) to quaternary materials (e.g., IGZO), and their characteristics vary depending on the composition ratio of materials^8–10^. The most widely known TAOS material is amorphous indium–gallium–zinc–oxide (a-IGZO). a-IGZO has excellent characteristics because of the composition of In^3+^ and Ga^4+^ ^8^. However, In and Ga are very rare elements on earth^11^. Studies are being conducted to find their nontoxic and abundant alternatives, and amorphous zinc–tin–oxide (a-ZTO) based materials are receiving considerable attention^12,13^. Recently, our group reported a-SZTO with improved stability by containing Si on the a-ZTO system^14,15^. Our study showed that Si atoms on the a-ZTO system inhibit oxygen vacancies. This is because they have a strong bonding strength with oxygen (799.6 kJ/mol)^15^. Many groups are conducting research on carrier enhancer elements and oxygen vacancy suppressor elements^3,5,9^. In general, TAOS materials generate electrons through oxygen vacancies^16^. The more oxygen vacancies, the better the electrical properties. However, it is very important to optimize oxygen vacancies because they can also act as traps. For a simple solution process, such as a sol-gel, oxygen vacancies should be controlled according to the composition ratio; however, in a vacuum process, such as a magnetron sputtering process, oxygen vacancies can be controlled by applying oxygen as the active gas^17,18^. Research on various deposition condition is being carried out, and the changes in electrical properties as well as energy bandgap have been analyzed in detail^19^. Among them, the oxygen partial pressure (*O*_*p*_) has a very important effect on the oxide semiconductor material. In the case of crystalline oxide semiconductors, As increase *O*_*p*_ the crystallinity improved but the energy bandgap decreases^20^. but in the case of TAOS, the energy bandgap increases according to *O*_*p*_^18^. Therefore, it is very important to analyze the correlation between *O*_*p*_ and energy bandgap. Zhao *et al*. analyzed Be_x_Mg_y_Zn_1-x-y_O as an oxide material having crystallinity, using the modified simplified coherent potential approximation method. The energy bandgap change according to the composition ratio of the material could be derived using the equation^21^. Recently, our group tried to derive the energy bandgap directly using methods such as high-resolution electron energy loss spectroscopy (HR-EELS), Kelvin probe force microscopy (KPFM), and ultraviolet photoelectron spectroscopy (UPS) to extract the energy bandgap^14,19^.

In this study, we have investigated the change in energy bandgap states systematically by using different *O*_*p*_ during the deposition to introduce different density of states (DOSs) inside the energy bandgap. In addition, the electrical and optical properties of a-SZTO thin films as TAOS materials have been characterized depending on the change in *O*_*p*_ during the deposition using the magnetron sputtering technique. It was observed that the energy bandgap systematically changed, and the performance also changed depending on the change in *O*_*p*_. In addition, we extracted the energy levels of the energy bandgap, Fermi level, and valence band maximum directly through HR-EELS, KPFM, and UPS. As a result, it was found that the change in *O*_*p*_ during the deposition directly affected the trap density within the TAOS thin film, and it was observed that the energy bandgap parameter can also be controlled by varying *O*_*p*_.

## Methods

### Device fabrication

The a-SZTO thin films with varying *O*_*p*_ were deposited on heavily doped p^+^-type silicon substrate (resistivity 0.001–0.002 Ω/cm), which has 100-nm thick SiO_2_ deposited through the thermal oxidation process, using radio frequency (RF) sputtering at room temperature. The fabricated TFTs have typical bottom gate and top source/drain electrode structures. Prior to deposition, the substrates were cleaned in acetone, methanol, and DI-water in an ultrasonication bath. The a-SZTO ceramic target was prepared by high purity (99.99%) powder mixtures of SiO_2_, SnO_2_, and ZnO. The Si was incorporated into the ZTO (Zn:Sn = 65:35) system at 1 wt.%. The sputtering conditions were: sputtering power of 60 W; deposition pressure of 3 mtorr; Ar partial pressure of 40 sccm; and *O*_*p*_ was varied among 0, 3, and 5 sccm. After the deposition, the a-SZTO thin films were annealed at 500 °C in ambient air for 2 h in a furnace. Then, the channels were patterned by conventional photolithography and wet-etching processes. The lift-off process was used to form the source/drain electrodes on the patterned channels. The source/drain electrodes were deposited with 10 nm and 40 nm of Ti and Al, respectively, using an E-beam and a thermal evaporator. The thicknesses of all fabricated a-SZTO thin films was fixed at 27 nm. The gate electrode and gate insulator of TFTs used heavily doped p+-Si and SiO_2_, respectively.

### Characterization

The crystallinity of a-SZTO thin films was analyzed through XRD (XRD, JP/SmartLab, Rigaku Co.). The transmittance of the thin films was measured using a UV-VIS-NIR spectrometer. The electrical properties were measured using a semiconductor parameter analyzer (HP-4145B, Hewlett-Packard Co.) and vacuum probe station (MS-TECH Co.). The O1s peaks of the thin films were measured through XPS, and fitting was performed using the general Gaussian–Lorentzian method. To calculate the energy bandgap parameters, each value was obtained using the following methods: *E*_*g*_ from HR-EELS (concentric hemispherical analyzer (CHA)-type, 23.5 eV energy resolution); valence band level from ((monochromatic He II); and Fermi level from KPFM.

## Results and Discussion

**Figure 1:**
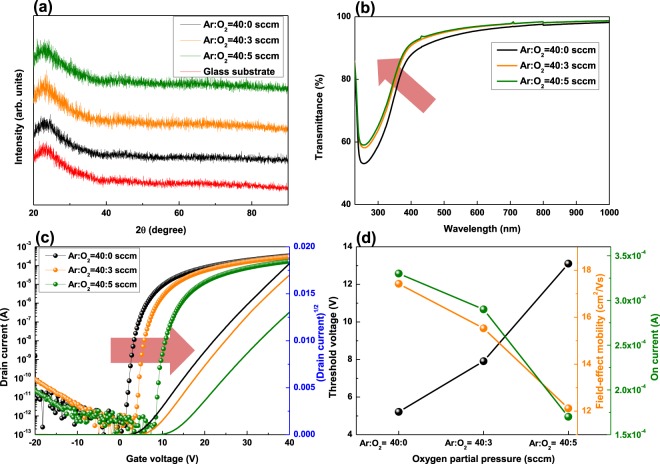
(**a**) X-ray diffraction pattern (XRD), (**b**) optical transmission spectra, (**c**) transfer characteristic and (**d**) electrical performances such as *V*_*th*_, *μ*_*fe*_, and *I*_*on*_ of a-SZTO TFTs, as function of oxygen partial pressure (*O*_*p*_).

Figure 1(a) shows the X-ray diffraction patterns (XRD) of a-SZTO thin film depending on *O*_*p*_. The XRD pattern clearly shows that no additional peaks are observed except for the broad peak located at approximately 23°. A broad peak was the peak output from the quartz glass substrate (corning 1737), used for the XRD measurement^22^. From the XRD results, it was confirmed that the a-SZTO thin film was amorphous, which did not exhibit crystallinity. In addition, the measurement results of transmittance with a variation in *O*_*p*_ is shown in Fig. 1(b). The transmittance of the visible region at 550 nm increased from 94.53% to 96.25% with an increase in *O*_*p*_, and the overall transmittance was above 90%. This is because of the wide energy bandgap of 3 eV or greater, which is a characteristic of the previously reported oxide semiconductor^3,9^. Fig. 1(c) shows the transfer curve of a-SZTO TFT depending on the change in *O*_*p*_. As *O*_*p*_ increased, the threshold voltage (*V*_*th*_) in the transfer curve systematically shifted to the positive direction from 5.21 to 13.10 V. *V*_*th*_ was obtained by applying the square-root to the drain-source current (*I*_*ds*_) value output from the transfer curve. *V*_*th*_ and other electrical characteristics are listed in Table 1. The field-effect mobility (*μ*_*fe*_) was obtained using the following equation^19^:

1 $${\mu }_{fe}=\frac{L{g}_{m}}{{{\rm{WV}}}_{{\rm{ds}}}{{\rm{C}}}_{i}},$$

where *g*_*m*_ is the transconductance, *C*_*i*_ is the oxide capacitance of the gate insulator, and *W* and *L* are the channel width and length, respectively. Electrical performance, such as *μ*_*fe*_, on-current (*I*_*on*_), subthreshold slope (*SS*), and on/off current ratio (*I*_*on/off*_) tends to decrease steadily as *O*_*p*_ increases. This is because when *O*_*p*_ increases during the deposition, the oxygen vacancy (*V*_*o*_) inside the thin film is suppressed^23^. Therefore, carrier concentration decreases with the suppression of *V*_*o*_, and electrical properties degrade. However, the suppression of these *V*_*o*_ can provide better stability^2,3^. In general, in oxide semiconductors, *V*_*o*_ provides electrons inside the thin film^16^. However, the optimization of the mobility enhancement and the stability is an important issue because *V*_*o*_ can also act as defected states^24,25^. Therefore, from Fig. 1(d) and Table 1, it can be clearly seen that, the oxygen atoms suppress the formation of *V*_*o*_, and the electrical properties degrade as *O*_*p*_ increases. In addition, we obtained the amount of trap state (*N*_*T*_) by using the *SS* value. The *SS* value and *N*_*T*_ have the following relationship^26^:

2 $${N}_{T}=(\frac{SSlog(e)}{kT/q}-1)\frac{{C}_{i}}{q},$$

where *SS* is the subthreshold slope, *k* is Boltzmann’s constant, *T* is the temperature, and *C*_*i*_ (3.45 × 10^−8^ F/cm^2^) is the unit gate capacitance. As *O*_*p*_ increased, *N*_*T*_ be decreased from 8.68 × 10^11^ cm^−2^ to 7.60 × 10^11^ cm^−2^. It was confirmed that the oxygen atom clearly decreased the *N*_*T*_ inside the a-SZTO thin film.

**Table 1:** Electrical performances of a-SZTO TFTs depending on *O*_*p*_.

Ar:O_2_ flow ratio (sccm)	*V*_th_ (V)	*I*_on_ (A)	*I*_on/off_	*µ*_FE_ (cm^2^V^−1^s^−1^)	*SS* (Vdecade^−1^)
40:0	5.21	3.3 × 10^–4^	3.3 × 10^9^	17.42	0.30
40:3	7.91	2.9 × 10^–4^	2.9 × 10^9^	15.53	0.29
40:5	13.10	1.7 × 10^–4^	1.7 × 10^9^	12.12	0.27

**Figure 2:**
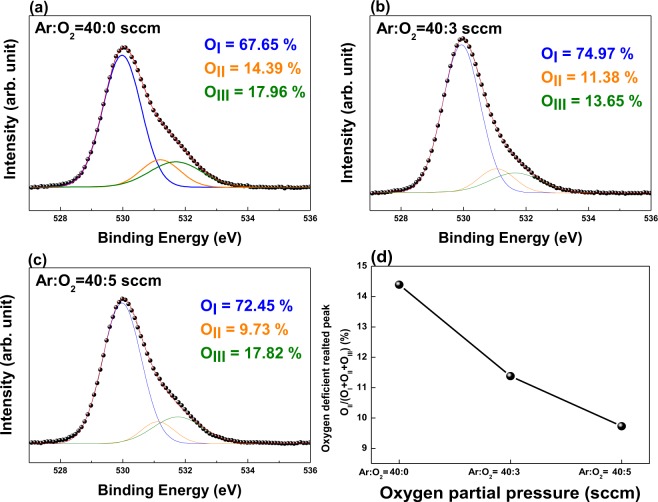
X-ray photoelectron spectroscopy (XPS) spectra of O 1 s peak of a-SZTO thin films depending on *O*_*p*_: (**a**) Ar:O_2_ = 40:0 sccm, (**b**) Ar:O_2_ = 40:3 sccm, and (**c**) Ar:O_2_ = 40:5 sccm. (**d**) The oxygen-deficient related peak (O_II_) in a-SZTO thin films with varying *O*_*p*_. Note that as the *O*_*p*_ increases, the O_II_ decreases.

Furthermore, we used X-ray photoelectron spectroscopy (XPS) to analyze the effect of the increase in *O*_*p*_ on the a-SZTO film. Figure 2(a–c) shows the XPS O1s peak of a-SZTO thin films with increasing *O*_*p*_. The binding energy of O 1 s peak was calibrated by taking C 1s as the reference at 284.25 eV. The O1s peak could be de-convoluted into three Gaussian–Lorentzian peaks: low binding energy (*O*_*I*_), middle binding energy (*O*_*II*_), and high binding energy (*O*_*III*_)^27^. The *O*_*I*_ peak can be attributed to metal–oxide bonding (M-O) and was observed at 530 ± 0.3 eV. The *O*_*II*_ peak is related to oxygen-deficient vacancies known as *V*_*o*_ and was observed at 531.2 ± 0.3 eV. The *O*_*III*_ peak is attributed to the surface oxygen owing to the hydroxide and was observed at 532.2 ± 0.3 eV^27,28^. From the XPS O1s results, it was observed that, the *O*_*II*_ peak (*O*_*II*_/*O*_*I*_ + *O*_*II*_ + *O*_*III*_), which is closely related to *V*_*o*_, decreased from 14.39% to 9.73% as *O*_*p*_ increased systematically as shown in Fig. 2(d). These results are the same as the decrease in trap density observed from the electrical properties. It is clear that the increase in *O*_*p*_ during the deposition suppresses *V*_*o*_ inside the a-SZTO thin film, thus degrading the electrical properties. Although the electrical properties degrade, the reduction of *V*_*o*_ can provide better stability. To confirm the stability enhancement, we measured the temperature stress (TS) and observed the relationship between the trap density in a-SZTO thin films and the change in *O*_*p*_.

**Figure 3:**
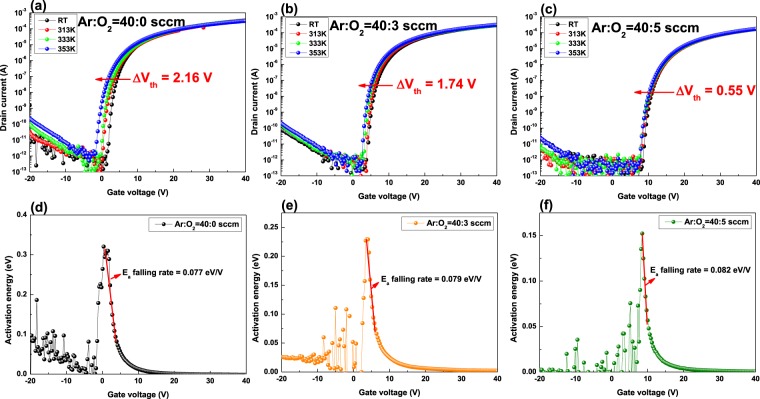
Variations in the transfer characteristics and activation energy (*E*_*a*_) of a-SZTO TFTs with varying *O*_*p*_ under the temperature stress from room temperature to 353 K: (**a**,**d**) Ar:O_2_ = 40:0 sccm, (**b**,**e**) Ar:O_2_ = 40:3 sccm, and (**c**,**f**) Ar:O_2_ = 40:5 sccm.

Figure 3(a–c) shows the TS results of a-SZTO TFT as *O*_*p*_ increases. The temperature dependency of TS was measured from room temperature to 353 K in intervals of 20 K. As *O*_*p*_ increased, the amount of shift of *V*_*th*_ (Δ*V*_*th*_) decreased from 2.16 to 0.55 V. Regardless of the increase in *O*_*p*_, it was confirmed that *V*_*th*_ moves toward the negative direction as temperature increases in the TS. It is expected that the thermal energy releases electrons in the trapped state inside the thin film with an increase in temperature^29^. We only applied the temperature parameter to analyze the effect on trap states inside the energy bandgap and did not add any additional stress, such as bias and illumination. In general, the operating mechanism of the oxide semiconductor occurs when electrons in the valence band are excited to the conduction band by an increase in energy^30^. However, electrons fill the trap states inside the energy bandgap^31^. This implies that, there are more electrons trapped inside the bandgap if the thin-film has more trap states. In addition, as the temperature increases, the trapped electrons receive a greater amount of energy. This allows the electrons in deep states to be excited into the conduction band as they absorb sufficient thermal energy. Therefore, a device with large trap states inside the energy bandgap will exhibit high Δ*V*_*th*_ value, which is not desirable. Here, we can see that the a-SZTO thin film deposited in pure Ar ambient shows the highest Δ*V*_*th*_ (2.16 V) value. It was also observed that Δ*V*_*th*_ decreases systematically as *O*_*p*_ increases. It is clear that the oxygen atom during the depositions fills the trap state associated with the *V*_*o*_ inside the a-SZTO thin film. The activation energy (*E*_*a*_) in the area below the subthreshold region was calculated using the TS results. The gate voltage (*V*_*gs*_) is plotted against *E*_*a*_ graphs from the TS results with a variation in *O*_*p*_ in Fig. 3(d–f), including the obtained *E*_*a*_ falling rate through the slope. The thermally activated drain current in the subthreshold region is obtained using the equation^29^

3 $${I}_{D}={I}_{D0}\cdot \exp \,(-\frac{{E}_{a}}{kT})$$

where *I*_*D0*_ is the pre-factor, *k* is Boltzmann’s constant, *T* is the absolute temperature, and *E*_*a*_ is the activation energy. The calculated *E*_*a*_ falling rate increased systematically from 0.077 eV/V to 0.082 eV/V as *O*_*p*_ increased. An increase in the falling rate implies a decrease in the channel and/or interface trap states^29,30^. In other words, in the graph of *E*_*a*_, the state can be changed more quickly if the amount of trap states is small for electrons to transit from the valence band to the conduction band. Therefore, as *O*_*p*_ increases, the amount of traps states in the energy bandgap decreases, and the *E*_*a*_ falling rate increases accordingly. Furthermore, to analyze the DOSs inside the a-SZTO thin film depending on *O*_*p*_, we used *E*_*a*_ to calculate DOSs based on the following equation^19^:

4 $${\rm{g}}({E}_{a})=-\frac{{\varepsilon }_{i}}{q{d}_{i}t\frac{d({E}_{a})}{d({V}_{gs})}}$$

where *ε*_*i*_ and *d*_*i*_ are the permittivity and thickness of the gate insulator, respectively, *t* is the thickness of the a-SZTO thin film (*t* = 27 nm), and *q* is the electron charge (1.602 × 10^−19^ C).

**Figure 4:**
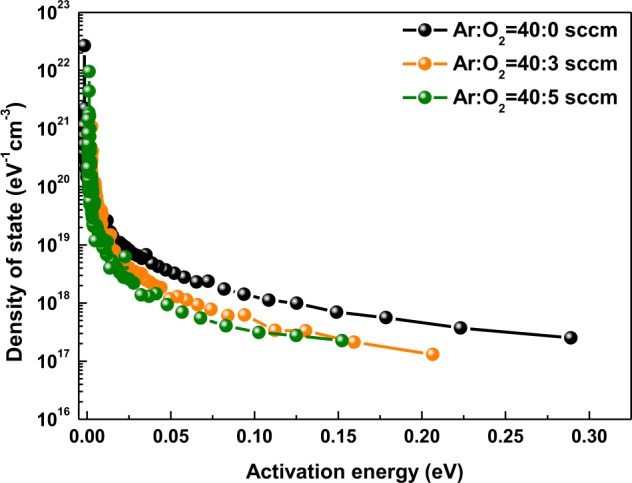
Calculated density of states (DOSs) versus activation energy (*E*_*a*_) for a-SZTO TFTs as function of *O*_*p*_.

Figure 4 shows the result of DOSs versus *E*_*a*_ calculated from *E*_*a*_ depending on *O*_*p*_. As *O*_*p*_ increases, the DOS tends to decrease systematically. This results from the oxygen atoms reducing the trap states that are closely related to the decrease of *V*_*o*_ inside the a-SZTO thin film during the deposition mentioned above. From the DOS results, we note that increasing *O*_*p*_ decreases the trap states in the shallow levels within the energy bandgap. As described above, the XPS O1s peak, electrical characteristics, and DOSs result from decreasing *V*_*o*_ as *O*_*p*_ increases. This implies that the energy bandgap can be adjusted indirectly according to *O*_*p*_ and, thus, the carrier concentration or the Fermi level can be controlled^23^. Therefore, to derive the energy bandgap directly, each energy level has been measured using ultra-violet photoelectron spectroscopy (UPS), Kelvin probe force microscopy (KPFM), and high-resolution electron energy loss spectroscopy (HR-EELS).

**Figure 5:**
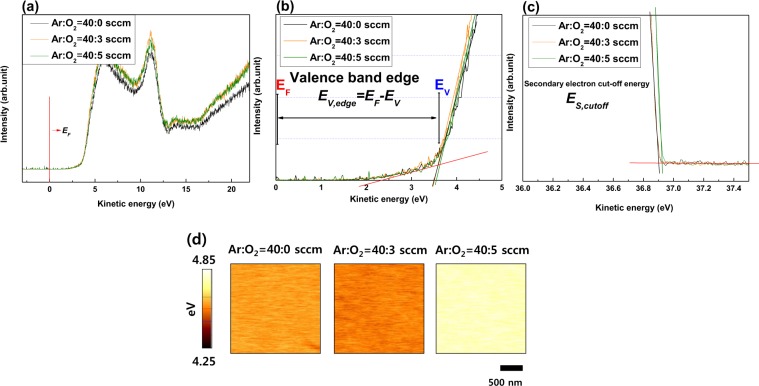
(**a**) Ultraviolet photoelectron spectroscopy (UPS) spectrum of the a-SZTO thin films depending on *O*_*p*_; (**b**) Expanded views of the low binding energy region for valence band edge (*E*_*F*_-*E*_*V*_), and (**c**) secondary electron cut-off energy with varying *O*_*p*_. (**d**) Kelvin probe force microscopy (KPFM) images of the a-SZTO as function of *O*_*p*_.

Figure 5(a–c) shows the UPS results, He (II) spectra of valence band edge (*E*_*V,edge*_), and secondary electron cut-off energy (*E*_*S,cutoff*_) of the a-SZTO thin films with varying *O*_*p*_. When a thin film is exposed to ultraviolet rays in a UPS measurement, it is known that a thin film surface and an impurity cause a chemical reaction to generate a surface dipole, which may cause a change in work function^32,33^. However, in order to produce a high-quality a-SZTO thin film according to *O*_*p*_, RF magnetron sputter was used in a high vacuum condition (<10^−6^ Torr) to minimize the surface impurities. In addition, considering the change of thin film by UV-induction, UPS was measured using separate specimens fabricated at the same time during sample fabrication. The points *E*_*V,edge*_ and *E*_*S,cutoff*_ depending on *O*_*p*_ were determined by the fitting method from the baseline. Based on the above results, the valence band level (*E*_*V*_) of the a-SZTO thin films can be directly calculated by the following equation^33^:

5 $${\rm{Valence}}\,{\rm{band}}\,{\rm{level}}\,({E}_{V},\,{\rm{eV}})=h{\rm{\upsilon }}-[{E}_{S,cutoff}-{E}_{V,edge}]$$

where $h{\rm{\upsilon }}$ is the energy of the monochromatic He (II) line emission at 40.813 eV, *E*_*S,cutoff*_ is secondary electron cut-off energy, *E*_*V,edge*_ is valence band edge. In addition, *E*_*V,edge*_ can be defined as the separation between the *E*_*V*_ and *E*_*F*_ as shown in Fig. 5(b). The calculated *E*_*V*_ shows little change from −7.490 eV to −7.510 eV with increasing *O*_*p*_. This indicates that the oxygen atoms during deposition do not directly affect the *E*_*V*_ region of the a-SZTO material. *E*_*F*_ according to the change of *O*_*P*_ measured by KPFM is shown in Fig. 5(d). The *E*_*F*_ contact potential difference was calibrated with respect to the reference of Pt/Ir (Tip *Φ* of 4.91 eV)^34,35^. Interestingly, it was observed that *E*_*F*_ has a large change of 0.1 eV or more in the a-SZTO thin films with increasing *O*_*p*_ (40:5 device). This large change means that oxygen atoms are more likely to suppress *V*_*o*_ as *O*_*p*_ increases, which means that the decrease in *V*_*o*_ in oxide semiconductors implies a decrease in the carrier concentration. In general, *E*_*F*_ is closely related to the carrier concentration. As the carrier concentration increases, *E*_*F*_ moves in the conduction band direction. Conversely, when the carrier concentration decreases, it moves in the valence band direction^36,37^. Therefore, *E*_*F*_ shifts toward the *E*_*V*_ direction due to the reduced carrier concentration.

**Figure 6:**
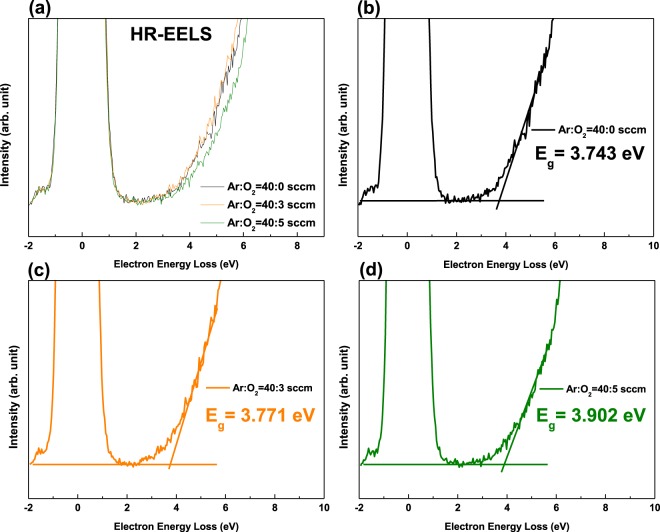
(**a**)High-resolution electron energy loss spectroscopy (HR-EELS) spectrum of the a-SZTO thin films depending on *O*_*p*_: (**b**) Ar:O_2_ = 40:0 sccm, (**c**) Ar:O_2_ = 40:3 sccm, and (**d**) Ar:O_2_ = 40:5 sccm. Note that as the *O*_*p*_ increases, the energy band-gap also increases.

**Figure 7:**
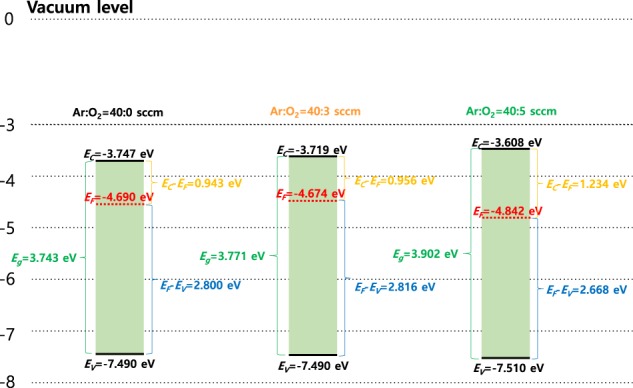
Calculated energy band-gap diagrams using energy position of conduction band, valence band, and Fermi level for a-SZTO thin films depending on *O*_*p*_. Note that the vacuum level is set to 0 eV.

Figure 6(a–d) shows the energy bandgap (*E*_*g*_) of the a-SZTO thin film with increasing *O*_*p*_ measured by using HR-EELS. The HR-EELS spectra were obtained by the transmitting electron beams with an energy of 1.5 keV. *E*_*g*_ was extracted by linear fitting the slope with respect to the baseline in the graph output from HR-EELS. As *O*_*p*_ increased, *E*_*g*_ showed an increasing tendency increase from 3.743 eV to 3.902 eV systematically. This is also consistent with the transmittance results shown in Fig. 1(b) above. We defined *E*_*g*_ using the energy bandgap parameters extracted from UPS, KPFM, and HR-EELS, as shown in Fig. 7. In addition, Table 2 summarizes the *E*_*g*_ values for each region. It was clearly observed that *E*_*g*_ increases systematically as *O*_*p*_ increases. Interestingly, it is particularly noteworthy that the area between the conduction band minimum (*E*_*C*_) and *E*_*F*_ varies greatly. It is observed that the increase in the overall *E*_*g*_ is mostly due to the expansion of the *E*_*C*_-*E*_*F*_ interval, which means that as *O*_*p*_ increases, the trap density in the *E*_*C*_-*E*_*F*_ interval, that is, the shallow level trap states, reduces significantly. The results of these measurements have been verified through DOS analysis, as shown above in Fig. 4. It is evident that the increase of *O*_*p*_ increases the *E*_*C*_-*E*_*F*_ region by filling the trap state at the shallow level. Additionally, it is observed to be the widest when *O*_*p*_ is 40:5. This large change is due to two main reasons: i) *E*_*F*_ shifts toward the *E*_*V*_ direction as the carrier concentration decreases, and ii) the reduction of the trap at the shallow^19,34^. Based on these results, it has been observed that the energy bandgap parameters of the oxide semiconductors can be controlled by the change in *O*_*p*_ during depositions resulting in the change of the *E*_*C*_-*E*_*F*_ interval significantly.

**Table 2:** Calculated energy band-gap parameters of a-SZTO TFTs depending on *O*_*p*_.

Ar:O_2_ flow ratio (sccm)	*E*_*g*_ (eV)	*E*_*F*_ (eV)	*E*_*C*_ (eV)	*E*_*V*_ (eV)	*E*_*C*_*-E*_*F*_ (eV)
40:0	3.743	−4.690	−3.747	−7.490	0.943
40:3	3.771	−4.674	−3.719	−7.490	0.956
40:5	3.902	−4.842	−3.608	−7.510	1.234

## Conclusion

In summary, we investigated the electrical properties and energy bandgap of the a-SZTO thin film by varying *O*_*p*_ during the deposition. Theoretical and experimental analyses were conducted to analyze the influence of *O*_*p*_ on the electrical characteristics of a-SZTO TFTs. An assumption was made that an increase in *O*_*p*_ can increase the energy bandgap, based on the transmittance measurements and the DOS results calculated via temperature stress. The increase in *O*_*p*_ during deposition was found to suppress the formation of oxygen vacancies within the a-SZTO thin film, which was mainly found to reduce the trap density of shallow levels. To observe the changes in the exact energy bandgap, HR-EELS, KPFM, and UPS were used to observe *E*_*g*_, *E*_*F*_, and *E*_*V*_, respectively. The increase in *O*_*p*_ observed a clear extension of the *E*_*C*_-*E*_*F*_ interval (0.943–1.234 eV) over the energy bandgap, which was found to be in good agreement with the DOS results. It was clearly observed that the change in *O*_*p*_ during deposition makes it possible to control the oxygen vacancy, which freely changes the energy bandgap of the oxide semiconductor. Therefore, the change of the a-SZTO thin film depending on *O*_*p*_ greatly affects the electrical characteristics and thermal stability of the TFT.
